# Prevalence of concurrent deep vein thrombosis in patients with lower limb cellulitis: a prospective cohort study

**DOI:** 10.1186/1471-2334-13-141

**Published:** 2013-03-19

**Authors:** Michael J Maze, Sean Skea, Alan Pithie, Sarah Metcalf, John F Pearson, Stephen T Chambers

**Affiliations:** 1 Department of Infectious Diseases, Christchurch Hospital, Christchurch, 8002, New Zealand; 2 Department of Radiology, Christchurch Hospital, Christchurch, 8002, New Zealand; 3 Department of Pathology, University of Otago, Christchurch, Christchurch, 8002, New Zealand; 4 Department of Public Health and General Practice, University of Otago, Christchurch, Christchurch, 8002, New Zealand

**Keywords:** Cellulitis, Erysipelas, Venous thromboembolism, Venous thrombosis

## Abstract

**Background:**

Lower limb cellulitis and deep vein thrombosis share clinical features and investigation of patients with cellulitis for concurrent DVT is common. The prevalence of DVT in this group is uncertain. This study aimed to determine the prevalence of deep vein thrombosis (DVT) in patients with lower limb cellulitis and to investigate the utility of applying the Wells algorithm to this patient group.

**Methods:**

Patients admitted with lower limb cellulitis prospectively underwent a likelihood assessment for DVT using the Wells criteria followed by investigation with D-dimer and ultrasonography of ipsilateral femoral veins as appropriate. Diagnoses of contralateral DVT or pulmonary embolism during admission were recorded.

**Results:**

200 patients assessed for DVT. 20% of subjects were high risk by Wells criteria. D-dimer was elevated in 74% and 79% underwent insonation of the affected leg. Ipsilateral DVT was found in 1 patient (0.5%) and non-ipsilateral VTE in a further 2 (1%).

**Conclusions:**

Deep vein thrombosis rarely occurs concurrently with lower limb cellulitis. The Wells score substantially overestimates the likelihood of DVT due to an overlap of clinical signs. Investigation for DVT in patients with cellulitis is likely to yield few diagnoses and is not warranted in the absence of a hypercoaguable state.

**Trial registration:**

ACTRN: 12610000792022 (https://www.anzctr.org.au/Trial/Registration/TrialReview.aspx?id=320662)

## Background

Cellulitis is a clinical diagnosis based on erythema, swelling and local tenderness of the skin and subcutaneous tissues accompanied by fever and malaise [1]. Cellulitis of the lower limb shares several clinical characteristics with deep vein thrombosis (DVT) and both have variability of clinical signs. These factors may lead to diagnostic uncertainty. This is particularly important in patients with clinically diagnosed cellulitis who are slow to respond to antimicrobials. A previous retrospective study in our institution indicated that approximately 15% of patients admitted to hospital with cellulitis underwent ultrasonography but the number of DVT detected was very low and only occurred in those with additional risk factors such as pelvic malignancy or injection drug use [2]. Other investigators have estimated the prevalence of DVT in patients with cellulitis or erysipelas. This has varied considerably from 1% to 15% [3-7]. Application of these results to clinical practice has been limited due to the absence of an assessment of the clinical likelihood of venous thromboembolism (VTE). The prevalence of DVT in this group is important because of the resources required for investigation for deep vein thrombosis, and treatment with anticoagulation has been proposed as an adjunct in patients with erysipelas due to the purported link [8].

There have been several studies outlining the value of clinical assessment for DVT. Goodacre et al. in a meta-analysis showed that individual clinical features are of limited value, but that a combination of variables increases the diagnostic utility [9]. The Wells score is a widely used clinical assessment tool using multiple clinical criteria, which when combined predict the probability of DVT [10]. The score ranges from −2 to 9, with a score of ≥2 designated as high risk. Populations previously studied for DVT have had prevalence estimated between 10-40%. In these studies the likelihood ratio of a high risk Wells score is 5.2 and 0.25 for those with low risk Wells scores [9-12]. In patients with skin and soft tissue infection there is a significant overlap of clinical signs with those of DVT, and the Wells score may be invalid if the prevalence of DVT is much lower in this population

The purpose of this study is to estimate the prevalence of DVT in patients with cellulitis or erysipelas and to determine the utility of applying the Wells algorithm to this patient group.

## Methods

This study is a prospective cohort study undertaken at the sole tertiary referral hospital in Christchurch, New Zealand. The trial was registered with the Australian New Zealand Clinical Trials Registry (ACTRN: 12-610000792022) https://www.anzctr.org.au/Trial/Registration/TrialReview.aspx?id=320662. The study was in compliance with the Helsinki Declaration and ethical approval was obtained from the Upper South B (New Zealand) Regional Ethics Committee (URB/10/02/006). Patients admitted under the adult internal medical service with a diagnosis of cellulitis or erysipelas of the lower limb were recruited within 48 hours of admission and investigated for DVT. Patients gave written informed consent prior to recruitment. The diagnosis of cellulitis was made on clinical grounds by an attending physician. Investigation for DVT was performed by calculation of a pre-test probability using the Wells score, with a score of ≥2 designated high risk, and D-Dimer assay (IL D-Dimer HS, Instrumentation Laboratory, Italy) using 250 ng/ml as the positive cut-off [9]. Ultrasonography of the leg was performed if the patient was high risk by the Well’s criteria or was low risk but had a positive D-dimer [9]. Ultrasonography was of the ipsilateral femoral and popliteal vein including a phasicity assessment of the common femoral vein [10]. If the patient was high risk on Wells criteria and had a positive D-dimer and negative ultrasound of the limb, they were followed up at 3 months and all subsequent diagnoses of DVT recorded.

It was calculated that recruitment of 203 patients would give a margin of error of +/− 3% if the prevalence of DVT was 5%. Due to logistical limitations, only 1 patient/day was recruited. When more than 1 case was admitted per day (range 0–3), the case for enrollment was randomly selected by die roll. Exact Clopper-Pearson 95% binomial confidence intervals around proportions were calculated using http://statpages.org/confint.html.

## Results

**Figure 1:**
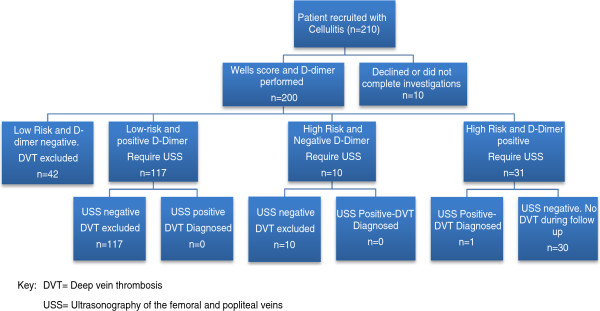
Flow chart for patients with cellulitis investigated for DVT.

The investigative algorithm is illustrated in Figure 1. Two hundred and ten cases were approached of whom 9 declined to participate. Of the remaining 201 subjects, 1 failed to complete the protocol. Patient characteristics are detailed in Table 1. Evaluation of the clinical risk of DVT by the Wells’ criteria categorized 41 (21%) patients at high risk and 151 (79%) at low risk of ipsilateral DVT. The prevalence of individual clinical characteristics is listed in Table 2.

**Table 1:** Patient characteristics


Age (mean/range)	62 years (17–96 years)
Gender	Male 110 (55%)
Ethnicity	Caucasian 182 (91%), Maori 12(6%) and Pacific People 6 (3%)
Fever (>38 degrees Celsius on admission)	111 (56%)
Pulse (median/range)	90 (55–149)
Blood Pressure (median/range)	131/70 (88-177/50-114)
Extent of cellulitis	Below knee 132 (66%), above knee 15 (7.5%), both above and below knee 53 (25.5%)
Skin and soft tissue changes	Blistering 34 (17%), associated ulcer 34 (17%), subcutaneous haemorrhage 30 (15%), associated wound 16 (8%), abscess formation 14 (7%)
Severity markers	Acute kidney injury 28 (14%),death during admission 8 (4%), bacteraemia 7 (3.5%) (S. aureus 4, beta haemolytic streptococci 3), Intensive care admission 3 (1.5%)
Co-morbidities	Heart disease 88(44%), diabetes 48(29%), chronic respiratory disease 56 (28%), chronic renal insufficiency 46(23%), central neurological disease or peripheral neurological disease affecting the lower limbs 34(17%), previous or active cancer 30 (15%), cirrhosis 5 (2.5%).
Anti-coagulant and anti-platelet medication	Anti-platelet therapy 70 (35%), and 14 (7%) anti-coagulant therapy 14 (7%).

**Table 2:** **Prevalence of individual Well**’**s score characteristics**


Active cancer	17 (8.5%)
Paralysis, paresis or recent plaster immobilization	12 (6%)
Recently bedridden >3 days/major surgery within 12 weeks	77 (38.5%)
Localized tenderness along the venous system	76 (38%)
Entire swollen leg	98 (49%)
Calf swelling >3 cm compared to that on asymptomatic side	152 (76%)
Collateral superficial veins	38 (18%)
Previous deep vein thrombosis	34 (17%)
Alternative diagnosis at least as likely as DVT	200 (100%)

Prevalence of suggestive clinical features such as leg swelling and venous tenderness was high and risk factors for thromboembolism were less common. Of the 77 who were recently bedridden or recently undergone major surgery, 27 had undergone surgery within 12 weeks and the remainder had been bedridden due to medical illness.

All patients underwent a D-dimer assay and 148 (74%) were elevated. 158 (79%) underwent insonation of their affected leg.

One of the 200 subjects (0.5%, 95% CI 0.01-2.7%) who completed the protocol was diagnosed with an ipsilateral DVT. The prevalence of DVT in the high risk group was 1/41(2.4%; 95% CI 0.6-12.8%) and 0/159 (0%; 95% CI 0.00-2.3%) in the low risk group, the difference is not significant using Fisher’s exact test (P=0.2), and with no cases in the low risk group the odds ratio is not finite. The subject with a DVT presented with 2 days of erythema of their calf and thigh and a fever of 39 degrees Celsius. This was preceded by 1 week of leg swelling. The subject's Wells score was 2, placing them in the high risk group, and their D-dimer was 1095ng/ml. Insonation of the affected leg found occlusive thrombus in the femoral vein.

In addition, one patient had a clinically silent contralateral DVT diagnosed when the wrong leg was scanned in error. One other patient was diagnosed with a symptomatic pulmonary embolus during her admission despite no ultrasonic evidence of DVT bilaterally.

## Discussion

This study demonstrates that despite high rates of clinically compatible signs, DVT prevalence in patients with cellulitis approximates that found in other studies of unselected hospital inpatients where prevalence of symptomatic venous thrombosis has been estimated at 0.6-0.8% [13,14]. The diagnosis in this study of a greater number of incidental venous thromboembolic events is in keeping with this.

The low prevalence of DVT in patients deemed high risk by the Wells’ criteria suggests that this evaluation tool is not suitable to this patient group. The overlap of clinical signs such as leg swelling reduces the specificity of this tool and the low prevalence adversely affects the positive predictive value of a “high risk” assessment. The very low prevalence of DVT contrasts with those in whom the Wells score was validated in which DVT was found in 27% of the high risk group and 4.3% of the low risk group [9]. All patients included in this study had 2 points deducted from the Wells score, due to the assessment that cellulitis was a more likely explanation for the clinical signs. If this was not done, the overestimation of risk would be much higher and the prevalence of DVT in the high risk group would be 0.6% (95% CI 0.02-3.7%).

In Wells’ study, 2 of 218 patients assessed as low risk and with a negative D-dimer were diagnosed with VTE within 3 months. On the basis of this result, it was asserted that ultrasonography can safely be omitted in this group [9,15]. The data from this study shows that in patients with cellulitis, prior to any risk stratification, DVT is just as unlikely.

The diagnosis of cellulitis was made solely at the discretion of the treating physician as there are no widely accepted criteria and cellulitis is diagnosed on clinical grounds [1]. It is therefore possible that other illnesses may be misdiagnosed as cellulitis. This uncertainty is an unavoidable aspect of management of cellulitis. There is limited application of the results of this study to those patients at very high risk due to the low number of these patients included. This primarily includes those with a hypercoaguable state, such as those with active malignancy. Anticoagulation will reduce the chance of DVT, but the small number of patients on anticoagulants were included as thrombosis can still occur, especially when anticoagulation is subtherapeutic. A large proportion of patients were taking aspirin but this does not substantially reduce venous thromboembolism [16]. The method of insonation of the femoral and popliteal veins was performed in accordance with current guidelines but only a single ultrasound was performed in the assessment of DVT. It is possible that calf DVTs were missed by this approach and clinical pathways recommend a repeat ultrasound at 5–7 days for those in the high risk group to ensure propagation of calf DVT into the femoral veins has not occurred [10]. No patients were diagnosed with DVT during the 3 months of follow up, which makes this unlikely to have an important affect on the estimated prevalence.

## Conclusion

In conclusion, DVT is a very uncommon occurrence in patients with cellulitis and current tools for assessing the likelihood of DVT overestimate the risk due to an overlap of clinical signs. Routine investigation for DVT in patients with cellulitis is likely to yield few diagnoses and is not warranted in the absence of a hypercoaguable state.

## Abbreviations

DVT: Deep vein thrombosis; VTE: Venous thromboembolism.

## Competing interests

The authors declare that they have no competing interests.

## Authors’ contributions

MM was involved in conception and design of the study, acquisition of data, analysis and interpretation of data and drafting the manuscript. SS, AP, SM, JP, SC were involved in conception and design of the study, analysis and interpretation of data and critically revised the manuscript. All authors read and approved the final manuscript.

## Pre-publication history

The pre-publication history for this paper can be accessed here:

http://www.biomedcentral.com/1471-2334/13/141/prepub
